# Dopaminergic Polymorphisms Associated with Time-on-Task Declines and Fatigue in the Psychomotor Vigilance Test

**DOI:** 10.1371/journal.pone.0033767

**Published:** 2012-03-16

**Authors:** Julian Lim, Richard Ebstein, Chun-Yu Tse, Mikhail Monakhov, Poh San Lai, David F. Dinges, Kenneth Kwok

**Affiliations:** 1 Cognitive Science Lab, Temasek Laboratories, National University of Singapore, Singapore, Singapore; 2 Department of Psychology, National University of Singapore, Singapore, Singapore; 3 Department of Economics, National University of Singapore, Singapore, Singapore; 4 Department of Pediatrics, National University of Singapore, Singapore, Singapore; 5 Department of Psychiatry, University of Pennsylvania Perelman School of Medicine, Philadelphia, Pennsylvania, United States of America; Centre national de la recherche scientifique, France

## Abstract

Prolonged demands on the attention system can cause a decay in performance over time known as the time-on-task effect. The inter-subject differences in the rate of this decline are large, and recent efforts have been made to understand the biological bases of these individual differences. In this study, we investigate the genetic correlates of the time-on-task effect, as well as its accompanying changes in subjective fatigue and mood. N = 332 subjects performed a 20-minute test of sustained attention (the Psychomotor Vigilance Test) and rated their subjective states before and after the test. We observed substantial time-on-task effects on average, and large inter-individual differences in the rate of these declines. The 10-repeat allele of the variable number of tandem repeats marker (VNTR) in the dopamine transporter gene and the Met allele of the catechol-o-methyl transferase (COMT) Val158Met polymorphism were associated with greater vulnerability to time-on-task. Separately, the exon III DRD4 48 bp VNTR of the dopamine receptor gene DRD4 was associated with subjective decreases in energy. No polymorphisms were associated with task-induced changes in mood. We posit that the dopamine transporter and COMT genes exert their effects by increasing dopaminergic tone, which may induce long-term changes in the prefrontal cortex, an important mediator of sustained attention. Thus, these alleles may affect performance particularly when sustained dopamine release is necessary.

## Introduction

Sustaining attention for a length of time is a prerequisite for performing many cognitive tasks. Failure to do so typically results in a vigilance decrement, or time-on-task (TOT) effect [1], [2], in which accuracy and/or reaction times degrade over the period of performance. This degradation is thought to be due to a weakening of top-down control due to high demands on neural resources [3], [4] as well as increased boredom and distractibility [5], in particular due to negative emotional stimuli [6], [7]. Lapses owing to fatigue and the TOT effect underlie many on-the-job errors that may in turn have serious real-world consequences [8], [9], thus providing the impetus to better characterize and understand the biological basis of this phenomenon.

Recently, investigators have observed that there is substantial inter-individual variability in the rate of TOT decline, with the most resilient subjects showing almost no decrement over a 20–30 minute period [10], [11]. It has been suggested that such resilience is trait-like, and that biomarkers may exist that differentiate resistant from vulnerable individuals. Indeed, a recent study by Lim et al., (2010) demonstrated that between subjects, resting-state cerebral blood flow in thalamus and middle frontal cortex is predictive of subsequent TOT decrements [11].

The existence of such biomarkers supports the hypothesis that time-on-task effects arise because resources in the brain are finite, and must be economically deployed during times of high attentional demand. If this is the case, individual differences in TOT vulnerability may be determined either by resource availability, or the ability of the neural system to tap into this reservoir. It has been proposed that dopamine (DA) may be one of the resources in question [10], [12]. To date, support for dopamine as being important to sustained attention has come largely from the literature on attention-deficit hyperactivity disorder (ADHD). ADHD is associated with prefrontal dysfunction [13], [14], and is treatable with the administration of DA agonists [15], [16]. Several common DA polymorphisms have been identified as risk alleles for this disorder, including the dopamine transporter gene DAT1 [17], [18], dopamine receptor genes [19], in particular DRD4 [20], catechol-o-methyl transferase (COMT) [21], which participates in the methylation and degradation of DA [22] and dopamine beta hydroxylase (DBH) [23], which converts dopamine to norepinephrine. The TaqIA polymorphism of another DA receptor, DRD2, has also been implicated in sustained attention deficits [24]. Behavioral genetics studies of these alleles suggest that they affect dopaminergic “tone” [25], which in turn moderates the integrity and stability of the sustained attention system. Findings from neuroimaging studies of vigilance and sustained attention lend further credence to this theory, as certain brain regions that distinguish between good and poor performers (e.g. the dorsolateral prefrontal cortex) [11], [26] are richly innervated by DA neurons [27].

In populations with ADHD, all of the alleles listed above (with the exception of the DRD2 TaqI polymorphism) have been linked to cognitive outcomes, in particular measures of executive attention such as response inhibition [20], [28]–[31]. The balance of evidence in this literature suggests that individuals with greater dopaminergic activity (e.g. through increased receptor binding affinity or reduced enzymatic activity) tend to have better cognitive performance, as measured by global task variables (i.e. accuracy/reaction time). In contrast, the genetic correlates of individual differences in attention in non-clinical populations are relatively poorly understood [12]. Some studies in healthy populations have replicated the findings cited above, showing, for example, that the COMT Met allele is associated with better executive control [32], though effects in the reverse direction have also been found [33].

Aside from the paucity of data on healthy individuals, studies of the effects of dopamine on cognition have focused largely on global variables, and have not examined the degradation of performance over time. As slope variables may measure a different facet of brain function than global variables, two competing hypotheses present themselves. Maintaining a steady level of functioning may depend on adequate dopamine signaling during the period of task performance in much the same way this benefits global outcomes. If so, we would expect individuals with alleles promoting higher levels of dopamine to show reduced TOT effects. Alternatively, TOT declines may be more greatly affected by efficient baseline levels of functioning, as suggested by previous fMRI data [11], in which case individuals with less dopaminergic activity may show less performance degradation. Our primary aim in this study, therefore, was to test these two hypotheses in order to determine the effects of dopaminergic polymorphisms on objective time-on-task decline as well as changes in subjective states in healthy individuals.

## Materials and Methods

### Ethics statement

This study was approved by the Institutional Review Board of the National University of Singapore, and all subjects provided written informed consent before participating in the study.

### Study procedure

A sample of N = 350 undergraduates, graduate students and staff members from the National University of Singapore were recruited through online advertising and word-of mouth. Subjects were pre-screened via a short telephone interview to ensure that they met all inclusion criteria. To qualify for the study, participants needed to be between 18 and 35 years of age, and ethnically Han Chinese. This latter criterion was chosen to decrease the possibility of artifacts owing to ethnic stratification, as Singapore is a racially diverse immigrant society. We excluded subjects who admitted to chronic physical or mental illness, had been diagnosed with a sleep disorder, or who were taking long-term medication. Subjects were also instructed to obtain a full night (>7 hours) of sleep for the 2 nights prior to the study, and to refrain from caffeine and alcohol for 6 hours prior to coming into the lab. All testing took place in the Cognitive Science Laboratory of Temasek Laboratories, Singapore.

On entering the lab, subjects first provided self-reports of their sleep history and alcohol/medication use over the previous 48 hours. They then provided self-ratings of several subjective states (fatigue, stress, anxiety, depression, sleepiness, motivation and energy) on a 9-point Likert-type scale. All questions took the form “How fatigued/sleepy/anxious etc. do you feel?” Following this, subjects were asked to remove their wristwatches and turn off their cell phones. They were then given instructions for performing the Psychomotor Vigilance Test (PVT) [34], and were provided an opportunity to practice the test for one minute. The practice period was kept relatively short to minimize pre-task fatigue, and as the PVT is known to have minimal practice effects [35]. After the practice, subjects were informed that they would have to complete a 20-minute version of the test [36], and that they should respond as quickly and accurately at all times to the stimuli even if they were feeling bored or tired. Subjects then performed the 20-minute PVT. To encourage subjects to exert maximum effort during the test, a research assistant was seated silently behind the participant at all times, as per standard test procedure. On completing the test, subjects immediately rated their subjective states again before completing the Pittsburgh Sleep Quality Inventory (PSQI) [37] and several other personality questionnaires. Personality data are not reported in this article. Finally, subjects donated genetic material via a saliva sample using the Oragene OG-500 collection kit (DNA Genotek, Ontario, Canada).

To check on the test-retest reliability of the PVT, a subset (N = 56) of participants returned to the lab one week after their initial session to complete the PVT a second time. Sleep history, caffeine and substance use were similarly controlled for this session.

Subjects were reimbursed $15 for their participation in the experiment ($25 for subjects who visited the lab twice).

### Behavioral data

The Psychomotor Vigilance Test (PVT) is a test of simple reaction time that is mentally demanding because of its high stimulus-load. The standard version of the test is 10 minutes in length; however, in order to elicit greater TOT effects, a 20-minute version was administered in this experiment. The Windows PennPVT (Pulsar Informatics, Philadelphia, PA) was used for stimulus presentation. During the test, subjects are required to monitor a small box subtending approximately 4.1 (width)×1.2 degrees (height) of visual angle for the appearance of a millisecond counter, whereupon they respond with a button press on the keyboard (space bar) as quickly as they can. The inter-stimulus interval of this counter lasts from 2 to 10 seconds (mean = 6 s), and RTs are uniformly distributed across this range of ISIs. Subjects are given 1 s after the counter stops to read their reaction time. Further details of the PVT and its psychometric properties can be found in references [36] and [38]. From each individual 20-minute bout, we measured the TOT effect using three different parameters. First, we extracted the linear slope of the reciprocal of reaction times (RRT). Additionally, we measured two parameters based on a least-squares fit to a two-component exponential function [39], which has been found to account for more of the variance in TOT than a simple linear fit. The equation used was:
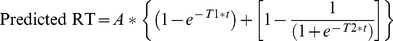
(1)where A is a multiplicative constant and t is time from test onset. Raw reaction times were averaged into one-minute bins prior to the application of this fit. We used parameters T_1_ and T_2_ as dependent variables for subsequent analyses; these parameters correspond respectively to a fast and slow decay in task performance.

### Genotyping methods

DNA was extracted from saliva samples, which were collected with Oragene DNA OG-500 tubes (DNA Genotek Inc., Ontario Canada) according to the manufacturer's protocol. Single nucleotide polymorphisms (SNPs) were analyzed by Sequenome MassArray genotyping; this analysis was performed at the Analytical Genetics Technology Centre, Princess Margaret Hospital, Toronto, Canada. The variable number of tandem repeats (VNTR) marker in the DAT gene was analyzed by PCR with ReddyMix™ PCR Master Mix (Thermo Scientific, Waltham, MA, USA). Primer sequences were: forward 5′-TGTGGTGTAGGGAACGGCCTG-3′, reverse 5′-CTTCCTGGAGGTCACGGCTCA-3′. Thermal protocol included activation step – 94°C for 5 min; 35 cycles of 94°C for 30 s, 55°C for 30 s, 72°C for 90 s; and final hold at 72°C for 5 min. The exon III DRD4 48 bp VNTR was analyzed by PCR with HotStar Plus DNA polymerase and Q-solution (Qiagen, Hilden, Germany). Primer sequences were: forward 5′- GCGACTACGTGGTCTACTCG -3′, reverse 5′- AGGACCCTCATGGCCTTG -3′. Thermal protocol included activation step – 95°C for 15 min; 40 cycles of 94°C for 30 s, 55°C for 30 s, 72°C for 40 s; and final hold at 72°C for 5 min. For both VNTR markers PCR products were visualized with electrophoresis in 1.5% agarose gel. The four SNPs analyzed were COMT Val158Met (rs4680), DBH Taq I (rs2519152), DRD2 Taq1A (rs1800497) and DRD4 -521C/T (rs1800955).

### Statistical analysis

Data analysis was performed using SPSS for Windows, Version 17.0 and MATLAB R2011B. Bivariate associations between objective and subjective data were assessed using Pearson's correlations. To reduce the possibility of Type I error in analyzing the genetic data, we restricted our search to six dopaminergic alleles, consisting of two VNTRs and four SNPs. We created two groups from each gene as follows: dopamine transporter gene (DAT1) VNTR (10/10 vs. 10/9, 9/9, 11/10 and other rare variants), DRD4 VNTR (2/2 and 2/4 vs. 4/4 and other rare variants), dopamine receptor genes DRD4 -521C/T (T/T vs. C/T and C/C), DRD2 TaqIA (any A1 allele vs. A2/A2), DBH Taq I (A/A vs. A/G and G/G) and COMT Val/Met (Val/Val vs. Val/Met and Met/Met). Overall univariate analysis of covariance (ANCOVA) was performed using, in turn, the three TOT measures (RRT slope, T_1_ and T_2_) as dependent variables, allele subgroups as between-subjects fixed factors, and subjective change in energy as a covariate. A separate ANCOVA was conducted using subjective change in energy as the dependent variable, allele subgroups as between-subjects fixed factors, and RRT slope as a covariate.

## Results

### Behavioral results

Data from 18 participants were excluded for the following reasons: not complying with instructions to obtain sufficient sleep (n = 11) and suspected non-compliance to task instructions (lapses and/or false alarms >20; n = 7). Examination of the PSQI revealed that a substantial number of individuals reported habitual poor sleep (PSQI>5; n = 72) as defined by the standards of Buysse et al. [37]. However, we found no correlation between PSQI scores and RRT slope, suggesting that this variable was not a confound in our results, and thus did not exclude subjects based on high PSQI scores. No subject had a PSQI score higher than 10. Our sample size after exclusions was therefore 332 (male = 172; mean age [SD] = 21.7 (1.7))

Overall, subjects performed the PVT well, as indicated by the low number of lapses (mean [SD] = 2.59 (3.28)) and false alarms (mean [SD] = 1.59 (2.17)). The average median response time over the entire sample was 264.3 ms (SD = 26.2). Inspection of the RRT slope showed that there was a decline in performance on average over the 20-minute period (mean [SD] = −0.026 (0.020)), as well as a substantial amount of interindividual variation around that mean (min = 0.0312; max = −0.089). More intuitively, raw reaction times were, on average, 14.2% slower during the last compared to the first four minutes of the task, with the worst performer showing a 101.4% increase, and the best a 12.9% decrease. Interestingly, and in agreement with previous studies of this phenomenon, 28 out of 332, or 8.4% of participants showed either no vigilance decrement, or a slight improvement in performance over time.

**Figure 1:**
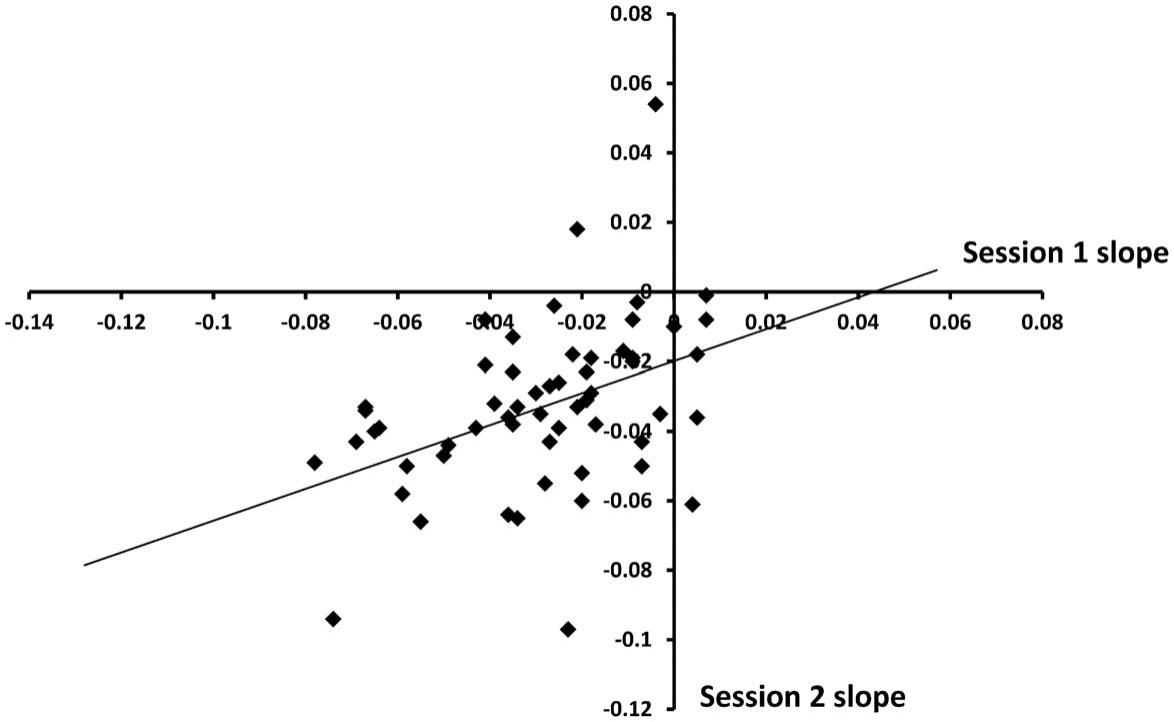
Inter-session reliability of time-on-task slope. Time-on-task slope is significantly correlated across two test sessions spaced one week apart (ICC_1,1_ = .54, *p*<.01). Subjects show a tendency to perform slightly worse in the second session.

We measured the stability of the time-on-task effect by inviting a subset of participants (n = 56) to undergo an identical second session of testing. Intra-class correlation coefficients were computed on these results to assess the test-retest reliability of TOT vulnerability. Between session (trait-like) reliability was significant, but moderate (ICC_1,1_ = .54, *p*<.01) A scatter plot of this correlation is shown in Figure 1.

In addition to the RRT slope, we obtained parameters T_1_ and T_2_ using the exponential fit in equation 1. While T_2_ was normally distributed, T_1_ showed a bimodal distribution, thus necessitating the use of bootstrapping in order to compare between-group means. We created a bootstrapped distribution using 10,000 draws from our original dataset, and calculated the difference of the adjusted means (accounting for subjective energy change) of T_1_ between each allele group to obtain an estimated p-value for each of these genes. Comparison of these p-values with those obtained from the straightforward ANCOVA revealed that they are extremely similar; we thus report results from the ANCOVA for simplicity.

### Subjective variables

In our main sample, performing the PVT resulted in significant changes in all subjective variables, with the largest differences in fatigue, sleepiness and energy (Table 1). Having made this observation, we aimed to reduce the dimensionality of these data by taking the difference between subjects' pre-task and post-task ratings, and subjecting these to factor analysis using a varimax rotation. Results of this analysis are reported in Table 2. Two components were found with eigenvalues of greater than 1 (Component 1: 2.307, 32.96% of variance; component 2: 1.522, 21.75% of variance). Fatigue and sleepiness loaded positively onto component 1, and motivation and energy loaded negatively onto this component. Stress, anxiety and depression loaded onto component 2. These factors may therefore be interpreted respectively as subjective change in energy (factor 1) and change in mood (factor 2). We created two composite indices of these factors by summing the relevant variables weighted by their respective factor loadings. Reliability for these scales for subjects who participated in two sessions was .52 (p<.01) and .54 (p<.01) respectively. These indices were used for all further statistical tests.

**Table 1:** Paired t-tests comparing subjective states before and after PVT performance.

Subjective variable	Pre-test average [SD]	Post-test average [SD]	*t*-value (*t*_330_)	Pre-post task correlation (*r*)
Fatigue	3.39 (2.07)	5.54 (2.13)	−18.82∧	.561
Stress	3.54 (1.76)	3.87 (1.71)	−4.26	.684
Anxiety	2.97 (1.60)	3.26 (1.69)	−3.97	.674
Sleepiness	3.28 (1.51)	4.99 (1.80)	−19.78	.564
Depression	2.18 (1.29)	2.52 (1.51)	−6.40	.778
Motivation	4.95 (1.40)	4.39 (1.34)	8.25	.624
Energy	5.28 (1.17)	4.17 (1.34)	17.94	.611

∧ degrees of freedom = 311 due to missing data.

All t and r values significant at *p*<.001.

**Table 2:** Factor analysis (with varimax rotation) of the change in subjective variables from pre- to post-task.

Variable	Factor 1 (subjective energy)	Factor 2 (mood)
Fatigue	**.659**	.112
Stress	.092	**.843**
Anxiety	−.033	**.883**
Sleepiness	**.689**	.136
Depression	.191	**.557**
Motivation	**−.702**	.016
Energy	**−.733**	−.101

Bold values indicate factor loaded on to by each variable.

We performed bivariate correlations to determine the relationship between objective performance declines and subjective mental states. RRT slope was correlated with the weighted index of subjective changes in energy (*r* = −.22, *p*<.001) but not changes in mood (*r* = −.033, n.s.). As a result, we chose to control for RRT slope when performing between-group tests on subjective changes in energy, and vice versa.

### Genetic data

Allele frequencies are presented in Table 3. We note that for the DRD4 VNTR, the 7-repeat allele, which is relatively common in Caucasian populations, is nearly absent among Han Chinese; instead, the 2-repeat allele is present in this population at approximately the same frequency. All candidate genes were found to be in Hardy-Weinberg equilibrium at *p*>.05.

**Table 3:** Allele counts and frequencies.

Genetic polymorphism	Genotype			Allele frequencies				Hardy-Weinberg equilibrium
DAT1 VNTR	9/9	9/10	9/11	10-repeat	9-repeat	11-repeat	Others	Yes (*p* = .91)
	1 (0.3%)	40 (12.1%)	0 (0.0%)	631 (91.2%)	42 (6.1%)	14 (2.0%)	5 (0.7%)	
	10/10	10/11	11/11					
	287 (87.0%)	12 (3.6%)	1 (0.3%)					
DRD4 VNTR	2/2	2/4	2/5	4-repeat	2-repeat	5-repeat	Others	Yes (*p* = .11)
	12 (3.8%)	89 (27.9%)	5 (1.6%)	519 (74.8%)	140 (20.2%)	14 (2.0%)	21 (3.0%)	
	4/4	4/5	5/5					
	206 (64.6%)	6 (1.8%)	1 (0.3%)					
DRD4 -521C/T	T/T	T/C	C/C		T allele	C allele		Yes (*p* = .09)
	150 (45.2%)	131 (39.5%)	48 (14.5%)		431 (67.0%)	227 (33.0%)		
DRD2 TaqIA	T/T	T/C	C/C		T allele	C allele		Yes (*p* = .99)
	112 (33.7%)	158 (47.6%)	55 (16.6%)		382 (58.8%)	268 (41.2%)		
COMT Val/Met	G/G	G/A	A/A		G allele	A allele		Yes (*p* = .99)
	183 (55.1%)	121 (36.4%)	20 (6.0%)		487 (75.2%)	161 (24.8%)		
DBH TaqI	A/A	A/G	G/G		A allele	G allele		Yes (*p* = .63)
	247 (74.4%)	70 (21.1%)	3 (0.9%)		566 (88.2%)	76 (11.8%)		

**Figure 2:**
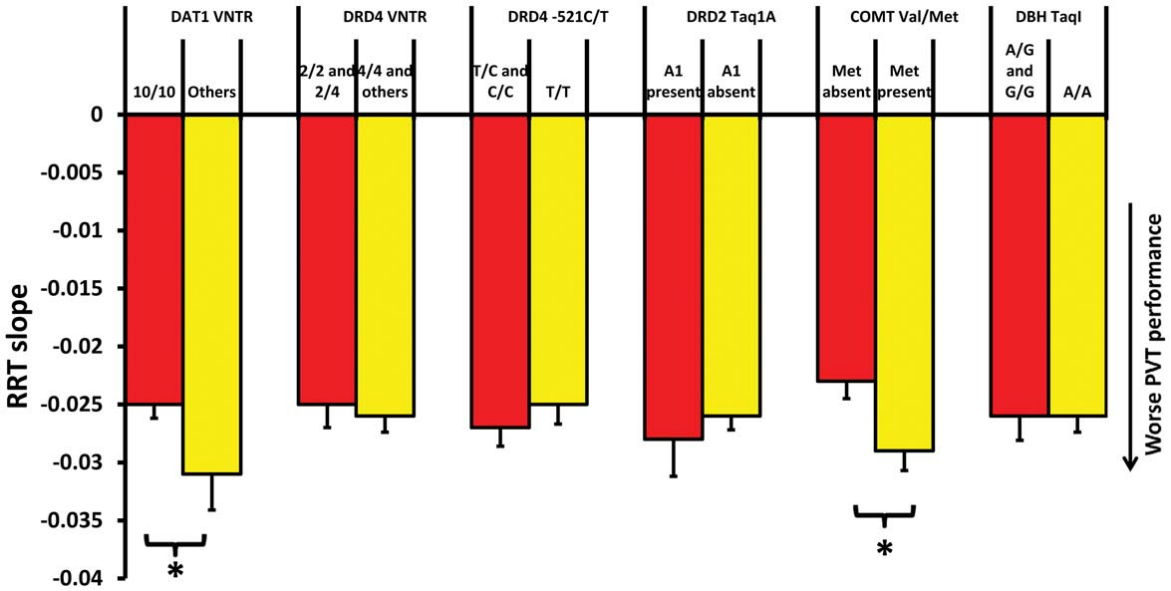
Average time-on-task slope by allele group. Means and standard errors for reciprocal reaction time slope in each allele group. * represents *p*<.05. Yellow bars represent allele groups thought to have greater dopamine availability.

**Figure 3:**
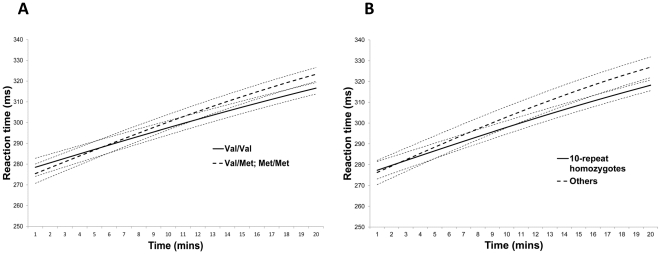
Average reaction time curves by allele group for DAT1 and COMT. Curves were plotted using predicted values calculated from average parameters A, T_1_ and T_2_ for each allele group. The thinner dotted curves represent the mean ± one standard error. Note that the curvature of the trends is only slight despite the use of an exponential equation; this is due to the bimodality of T_1_, for which large values cause the curve to approach linearity. Panel A: COMT allele groups. Panel B: DAT1 allele groups.

We first examined the genetic data using a univariate approach to determine which of our key outcome variables differed depending on genotype group. These outcome variables were RRT slope and the indices for subjective change in energy and mood. None of the covariate by group interactions was significant at the *p*<.05 level. Means and group sizes for each allele group are shown in Table S1. After controlling for subjective change in energy, we found that the omnibus test was significant for RRT slope (F_7,308_ = 4.93, p<.001) and T_2_ (F_7,308_ = , p = .001), but not T_1_. In the analysis of the linear RRT slope, DAT1 VNTR (F_1,325_ = 4.59, *p*<.05) and COMT Val/Met (F_1,325_ = 5.01, *p*<.05) polymorphisms were significantly associated with time-on-task declines (Table 4; Figure 2). In both these cases, genotypes thought to result in greater dopaminergic tone (the 10-repeat present group for DAT1 and the Met-allele absent group for COMT) were associated with steeper declines in performance over time. Effect sizes (partial η^2^) for these alleles were in the small-medium range (.015 and .016 respectively). The difference between allele groups for DRD4 approached significance (F_1,325_ = 3.87, *p* = .051) Using the parameters from the exponential fit, we found that DAT1 and COMT were associated with the slow decay parameter (T_2_) but not the fast decay (T_1_) (Table 5). To better visualize this difference, we plotted average curves for each allele group using the estimated subject parameters; these are displayed for comparison in Figure 3.

**Table 4:** Univariate ANCOVA of the effects of dopamine alleles on RRT slope.

Source	Type III SS	df	Mean square	F	Significance	Partial η^2^
Full model	0.013	7	0.002	4.93	<.001	.103
Intercept	0.029	1	8×10^−6^	76.67	<.001	.203
**Factors**						
DRD4 VNTR	0.001	1	0.001	3.87	.051	.013
DAT1 VNTR	0.002	1	0.002	**4.59**	.033	.015
DRD4 -521 C/T	0.001	1	0.001	1.64	.201	.005
DRD2 Taq1A	0.000	1	0.000	1.07	.302	.004
COMT Val/Met	0.002	1	0.002	**5.01**	.026	.016
DBH TaqI	8.5×10^−5^	1	8.5×10^−5^	0.22	.684	.001
**Covariate**						
Subjective change in energy	0.008	1	0.008	21.5	<.001	.067

Values in bold text are significant at *p*<.05.

**Table 5:** Univariate ANCOVA of the effects of dopamine alleles on exponential time-on-task parameters.

	T_1_						T_2_					
Source	Type III SS	df	Mean square	F	Significance	Partial η^2^	Type III SS	df	Mean square	F	Significance	Partial η^2^
Full model	2990.1	7	427.2	1.32	.211	.028	0.086	7	0.012	3.31	.001	.077
Intercept	22722.4	1	22722.4	66.61	<.001	.181	0.159	1	0.159	46.28	<.001	.133
**Factors**												
DRD4 VNTR	365.2	1	365.2	1.07	.302	.004	0.007	1	0.007	2.27	.151	.007
DAT1 VNTR	70.1	1	70.1	0.11	.651	.001	0.020	1	0.020	**6.36**	.017	.019
DRD4 -521 C/T	59.6	1	59.6	0.28	.676	.001	0.006	1	0.006	2.18	.185	.006
DRD2 Taq1A	38.1	1	38.1	0.06	.739	.000	0.002	1	0.002	0.78	.400	.002
COMT Val/Met	704.5	1	704.5	2.15	.152	.007	0.018	1	0.018	**5.17**	.024	.017
DBH TaqI	0.1	1	0.1	0.01	.984	.000	0.008	1	0.008	2.36	.131	.008
**Covariate**												
Subjective change in energy	1401.2	1	1401.2	4.78	.044	.013	0.031	1	0.031	9.82	.002	.029

Values in bold text are significant at *p*<.05. As T_1_ was not normally distributed, we calculated t-values for between-group comparisons on adjusted means for this variable using a bootstrapping method (see text for details); as these results were highly similar to the ANCOVA results, the latter are reported here for simplicity.

Given the effects of DAT1 and COMT on TOT, we wished to further probe the effect of these alleles on other common PVT parameters. Accordingly, we extracted the number of lapses (responses>500 ms) and false alarms, and computed a metric of attentional stability [40], the intra-individual coefficient of variation (ICV), by dividing the SD of reaction times by the mean [41]. As the distribution of ICV showed significant skewness, we normalized it by taking its inverse before performing further statistical tests on this variable. RRT slope was significantly correlated with lapses (*r* = −.71, *p*<.001), false alarms (*r* = −.33, *p*<.001) and ICV (*r* = −.39, *p*<.001). After controlling for subjective change in energy, we found no differences between genotype groups in either DAT1 or COMT in any of these variables.

When confronted with a vigilance task, it is possible that subjects may withhold effort at the beginning of the trial so as to conserve resources, and thus better maintain performance. As part of the study setup, we took steps to mitigate this issue by instructing participants to exert maximum effort at all times, and by having an experimenter observe the subject throughout the study. We checked on this potential confound by correlating RRT slope with mean reaction time from the first minute of the PVT, and found a small but significant positive correlation between them (r = .13, *p*<.05). Having discovered this, we again looked for differences in genotype group based on this dependent variable, but found no differences in DAT1 or COMT. Furthermore, adding this variable as a covariate to the ANCOVAs reported above does not materially change our main results. Thus, the genetic differences observed seem to be exclusively related to TOT performance decrements.

**Figure 4:**
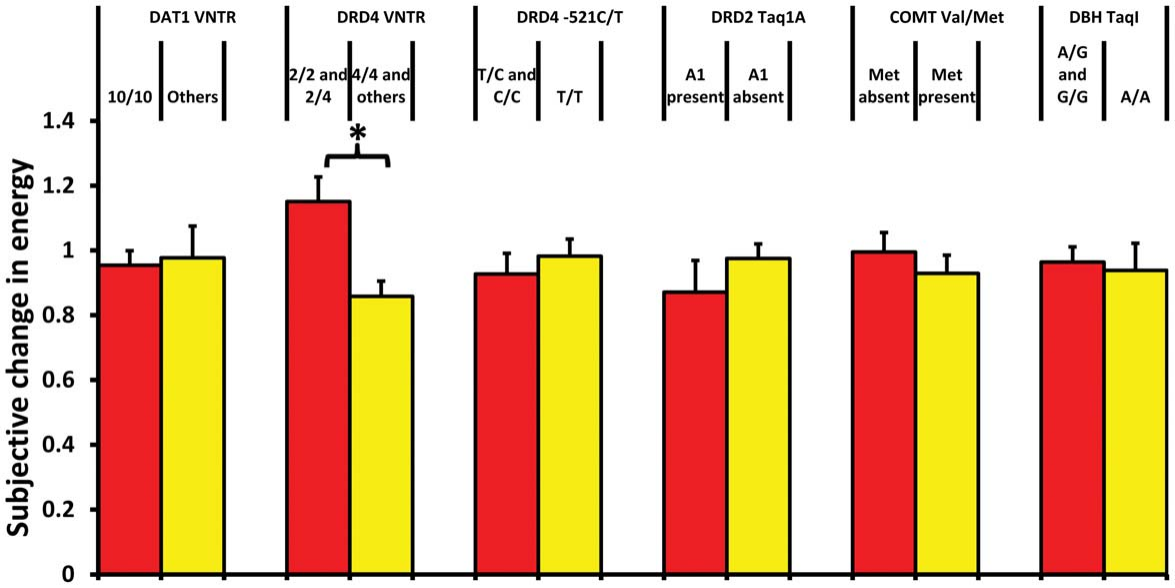
Average subjective change in energy by allele group. Means and standard errors for average subjective energy change in each allele group. * represents *p*<.05. Yellow bars represent allele groups thought to have greater dopamine availability.

For the subjective data, the overall effect of the six dopamine polymorphisms was also significant (F_7,308_ = 3.43, p = .002); however, only the DRD4 VNTR (F_1,325_ = 13.71, *p*<.001, η^2^ = .044) was significantly related to changes in energy across the duration of the task (Table 6; Figure 4) after controlling for RRT slope. No significant associations were found between any of our candidate genes and subjective changes in mood.

**Table 6:** Univariate ANCOVA of the effects of dopamine alleles on subjective change in energy.

Source	Type III SS	df	Mean square	F	Significance	Partial η^2^
Full model	11.9	9	1.70	3.43	.002	.074
Intercept	48.2	1	48.2	97.37	<.001	.244
**Factors**						
DRD4 VNTR	6.78	1	6.78	**13.71**	<.001	.044
DRD1 VNTR	0.12	1	0.12	0.24	.624	.001
DRD4 -521 C/T	0.01	1	0.01	0.01	.905	.000
DRD2 Taq1A	1.19	1	1.19	2.40	.122	.008
COMT Val/Met	0.17	1	0.17	0.33	.564	.001
DBH TaqI	0.15	1	0.15	0.30	.585	.001
**Covariate**						
RRT slope	4.40	1	4.40	8.90	<.003	.029

Values in bold text are significant at *p*<.05.

## Discussion

The results of this study demonstrate links between several functional dopaminergic alleles and the propensity to both decline in performance and feel mentally fatigued. Specifically, our data suggest that the dopamine transporter, DAT1, as well as COMT, may have an impact on the rate at which the vigilance decrement occurs, and that the dopamine receptor DRD4 may be related to subjective declines in mental energy. This is one of the first demonstrations that these polymorphisms play such a role in attention in non-clinical populations.

For the two genes that were associated with TOT, alleles that typically confer risk of poorer cognitive performance (i.e. the Val/Val allele [21], [42] and the 10-repeat allele of the DAT1 VNTR [17], [18], [28]) were protective against TOT declines in our sample, although selective studies on healthy populations have also found effects in the direction indicated in this work [43]. The two alleles in question are thought to increase the availability of DA to post-synaptic neurons, thus promoting activity in striatal and prefrontal regions implicated in stable attentional performance. Thus, our results argue against the hypothesis that global variables such as mean reaction time and the slope variable of time-on-task are affected in the same way by dopaminergic activity

The direction of the dopamine effect in this study may be best explained by the tonic-phasic model of dopamine regulation. In a seminal set of papers, Grace and colleagues [25], [44] described this dissociation, and showed that dopamine exerts an influence on post-synaptic activity via two separate but related mechanisms. First, phasic DA release is triggered when bursts of action potentials are fired in response to environmental stimuli and reach DA axon terminals [45]. This release leads to a large and rapid rise in intrasynaptic dopamine; however, this rise is also highly transient due to the immediate action of the DA transporter, which causes reuptake of DA into pre-synaptic terminals [46]. In contrast, tonic DA activity refers to spontaneously occurring single spikes in DA neurons [47] which determine the baseline levels of DA concentration in extracellular space [48].

In the long-term, the tonic and phasic DA systems are not independent. For instance, stimulants such as cocaine block reuptake of DA from the synaptic cleft [49], allowing increased leakage of DA into extracellular space. Abusers of these stimulants tend to have an increase in tonic DA activity, and a subsequent down-regulation of the post-synaptic DA system over time (for a comprehensive review, see [23]). Furthermore, it has been demonstrated that individuals with relatively low dopaminergic tone also have larger phasic release of DA in response to an external stimulus (e.g. nicotine) [50]. These results may have implications for cognitive functioning; for instance, a model of dopamine function in PFC has shown that tonic and phasic evels of DA interact to determine optimal performance on a task – phasic increases are most beneficial in PFC when basal (tonic) DA activity is low [51].

As mentioned previously, alleles associated with greater DA availability are usually found to be of benefit in a range of cognitive tasks. However, our data suggest that slope variables may be affected much more greatly by DA tone than phasic DA release, thus explaining the inferior performance of individuals with DA-promoting alleles. Differences between DAT1 and COMT genotype groups in this sample were found only for TOT variables, and not lapses, false alarms or reaction time variability, despite the fact that these variables are highly inter-correlated. We also found evidence that some subjects were able to ameliorate their level of TOT decline by withholding effort (possibly unconsciously), as mean first-minute reaction times were positively correlated with RRT slope. Nevertheless, DAT1 and COMT genotype groups did not differentiate performers on this variable either. We therefore speculate that individuals with chronically high DA availability may more quickly exhaust the benefits of greater phasic release when longer-term attentional effort is required, due to the long-term plastic changes in DA neurons in the striatum and PFC described above. These effects may be especially prominent when elicited by the PVT, which is a task with consistently high signal load and attentional demand.

The assertion that tonic, resting levels of DA may influence TOT changes is consistent with data from a recent study in which Lim et al. [11] used fMRI to measure the baseline and task-related activity of a group of young subjects as they performed the Psychomotor Vigilance Task (PVT). The authors found that pre-task resting cerebral blood flow in the thalamus and middle frontal gyrus (MFG) predicted the extent of subsequent performance decline, with higher MFG activity at rest correlated with greater TOT decrements. Taken together with our current genetic data, we speculate that this higher MFG resting perfusion may be related to greater spontaneous firing rates which result from higher DA tone. Indeed, negative correlations have been found between striatal DAT density and regional cerebral blood flow in frontal cortex [52], providing *in vivo* support for this claim. This view is also consistent with the notion that fMRI activity may represent an endophenotypic trait [53], [54] that mediates between genetic differences and behavioral phenotypes.

While both DAT1 and COMT were found to have an effect on PVT performance in this study, presumably via the modulation of levels of extracellular DA, we note that these molecules likely influence this phenotype via different sites in the brain. COMT acts to degrade DA primarily in PFC, with a ∼40% difference in enzyme activity between the Val and Met alleles [55]. Furthermore, COMT is significantly less active in the striatum than PFC, as DA persists in extracellular space for a much longer time in the PFC [56]. In contrast, the DA transporter has a much larger effect on reuptake in the ventral striatum than in frontal cortex [57]. Hence, DAT1 may exert its influence on attention indirectly, possibly by modulating levels of motivation during the task. Further research is needed to test the more fine-grained effects of these two polymorphisms on TOT decrements.

### Fast vs. slow decay

By decomposing individual subject curves into two components using a set of exponential functions, we found that DAT1 and COMT polymorphisms were associated with differences in the slow, but not the fast decaying component. Effect sizes of genotype on the slow component were, in fact, slightly larger than for the simple linear fit. These data suggest that dopamine exerts its effects on longer-term TOT decay, which lends credence to the hypothesis that the direction of the effects observed in this experiment (i.e. COMT_Met_<COMT_Val_; DAT1_10-repeat absent_<DAT1_10-repeat present_) may only be observable when the dependent measure is a slow-evolving process.

### Subjective changes

As expected, performing the PVT for 20-minutes resulted not only in robust TOT decrements, but also significant declines in energy and mood. This is consistent with findings from previous studies which suggest that vigilance tests are a resource-demanding form of mental work [3], [4]. A factor analysis showed that energy and mood changes loaded onto separate scales, suggesting that different biological pathways may underlie these subjective changes. These factors correspond well with a two-system model of activation (energetic and tense arousal) proposed by Thayer [58], adding support for their validity. Moreover, in the current dataset, we found that objective TOT declines were correlated with changes in energy, but not mood, indicating that the former scale more closely reflects the phenomenology associated with mental workload.

Interestingly, subjective changes in energy were strongly associated with the DRD4 polymorphism; subjects with at least one copy of the 2-repeat allele tended to show a greater subjective change in energy over time. To our knowledge, this is the first association of the allele with this effect, although previous work has implicated DAT1, COMT and DRD2 in mental fatigue [59], and the role of dopamine in maintaining an energetic state has been extensively discussed [60]. Experimentally, catecholamine depletion is known to decrease energy and vigor, and increase sleepiness, fatigue and sedation [61]. Reproducing and explaining the effect of DRD4 on subjective mental fatigue represents a promising avenue for ameliorating this problem in real-world situations.

Finally, significant increases in anxiety, stress and depression represent a separate problem that is putatively caused by high mental workload. Our data suggested that changes in mood are not directly associated with TOT; nevertheless, they represent an undesirable side effect that may lead to other negative consequences. Further research is needed to characterize how these changes are instantiated in the brain, and how they might affect behavior and performance.

### Limitations

The current study has a small number of limitations. First, subjects were asked to abstain from caffeine, which may have caused some regular users to experience a withdrawal effect. However, data collected from other studies by the first author indicate that caffeine usage in Singapore students is low, and we thus suggest that any such effects were relatively minor. Second, and more importantly, we note that our sample size was moderate for a study of this nature, and that the effects observed were in the small to medium range, exposing us to the possibility of Type I error. Nevertheless, we count two points in our favor. First, we had strong *a priori* justification based on the previous clinical literature that the alleles under investigation are involved in sustained attention, and tested only a small number of these candidate genes. Second, more than one of the comparisons performed returned a significant result in the same direction, and as these form part of a single system, are unlikely when taken together to be spurious findings.

We note that the reliability of the TOT effect, as is typical with change scores, is only in the moderate range (.54), as are the intra-class correlations of the subjective measures used in this paradigm. This is not unexpected, as difference or slope scores by their nature have lower reliability than measures of central tendency [62]. We believe that the relatively lower reliability of change scores speaks more to the method of their computation than against the trait-like nature of TOT vulnerability; however, we recommend that large sample sizes be used when studying this phenomenon in the future to ensure that results are valid and reproducible.

### Conclusion

An increasing amount of evidence points to TOT vulnerability as being the result of the cortical attention system drawing on a brain-limiting resource that is determined by resting levels of neuronal activity. Our genetic data strengthen the case that one of the resources in question may be dopamine, and that these individual differences may be more highly related to tonic brain functioning than phasic task-related activity. We further demonstrate that TOT vulnerability is associated with changes in energy but not mood, and that DRD4 is robustly related to the feelings of energy depletion. The results of this study have implications for the emerging field of neuroergonomics [63], [64], and add to our knowledge of the biological basis of mental “work” and its fatiguing effects.

## Supporting Information

Table S1 **Mean [SE] scores for time-on-task and subjective energy change by allele group.**
(DOCX)
