# Supplementary material for: Dopaminergic Polymorphisms Associated with Time-on-Task Declines and Fatigue in the Psychomotor Vigilance Test
Source: PLoS One. 2012 Mar 16;7(3):e33767. doi: 10.1371/journal.pone.0033767 (PMC3306301; doi:10.1371/journal.pone.0033767)
Supplement: Table S1 — Mean [SE] scores for time-on-task and subjective energy change by allele group. (DOCX) [file pone.0033767.s001.docx]

**Table S1.** Mean [SE] scores for time-on-task and subjective energy change by allele group

| **Genetic polymorphism** | **Genotype groups** | **Group size** | **RRT slope** | **Subjective change in energy** |
| --- | --- | --- | --- | --- |
| DAT1 VNTR | 10/10 | 272 | -0.025 (0.0012) | 0.954 (0.045) |
|  | 10/9, 9/9, 11/10, others | 56 | -0.031 (0.0031) | 0.977 (0.098) |
| DRD4 VNTR | 2/2 and 2/4 | 114 | -0.025 (0.0020) | 1.151 (0.076) |
|  | 4/4 and others | 216 | -0.026 (0.0014) | 0.858 (0.047) |
| DRD4 -521C/T | T/T | 150 | -0.025 (0.0017) | 0.927 (0.064) |
|  | T/C and C/C | 182 | -0.027 (0.0016) | 0.982 (0.053) |
| DRD2 Taq1A | A1 present | 55 | -0.028 (0.0032) | 0.871 (0.098) |
|  | A1 absent | 270 | -0.026 (0.0012) | 0.975 (0.045) |
| COMT Val/Met | Met present | 141 | -0.029 (0.0017) | 0.995 (0.060) |
|  | Met absent | 183 | -0.023 (0.0015) | 0.929 (0.056) |
| DBH TaqI | A/A | 246 | -0.026 (0.0014) | 0.964 (0.047) |
|  | A/G and G/G | 74 | -0.026 (0.0021) | 0.938 (0.084) |

Values that are more negative for RRT slope represent worse performance.
